# Deficient tRNA posttranscription modification dysregulated the mitochondrial quality controls and apoptosis

**DOI:** 10.1016/j.isci.2024.108883

**Published:** 2024-01-12

**Authors:** Yunfan He, Gao Zhu, Xincheng Li, Mi Zhou, Min-Xin Guan

**Affiliations:** 1 Center for Mitochondrial Biomedicine, the Fourth Affiliated Hospital, Zhejiang University School of Medicine, Yiwu, Zhejiang, China; 2 Institute of Genetics, Zhejiang University International School of Medicine, Hangzhou, Zhejiang, China; 3 Center for Genetic Medicine, Zhejiang University International Institute of Medicine, Yiwu, Zhejiang, China; 4 Zhejiang Provincial Key Laboratory of Genetic & Developmental Disorders, Hangzhou, Zhejiang, China; 5 Key Lab of Reproductive Genetics, Ministry of Education of PRC, Zhejiang University, Hangzhou, Zhejiang, China

**Keywords:** Properties of biomolecules, Molecular physiology, Cell biology

## Abstract

Mitochondria are dynamic organelles in cellular metabolism and physiology. Mitochondrial DNA (mtDNA) mutations are associated with a broad spectrum of clinical abnormalities. However, mechanisms underlying mtDNA mutations regulate intracellular signaling related to the mitochondrial and cellular integrity are less explored. Here, we demonstrated that mt-tRNA^Met^ 4435A>G mutation-induced nucleotide modification deficiency dysregulated the expression of nuclear genes involved in cytosolic proteins involved in oxidative phosphorylation system (OXPHOS) and impaired the assemble and integrity of OXPHOS complexes. These dysfunctions caused mitochondrial dynamic imbalance, thereby increasing fission and decreasing fusion. Excessive fission impaired the process of autophagy including initiation phase, formation, and maturation of autophagosome. Strikingly, the m.4435A>G mutation upregulated the PARKIN dependent mitophagy pathways but downregulated the ubiquitination-independent mitophagy. These alterations promoted intrinsic apoptotic process for the removal of damaged cells. Our findings provide new insights into mechanism underlying deficient tRNA posttranscription modification regulated intracellular signaling related to the mitochondrial and cellular integrity.

## Highlights

• tRNA^Met^ 4435A>G mutation impaired the assembly and activity of OXPHOS complexes
• Mitochondrial dysfunctions led to imbalance of mitochondrial dynamics toward fission
• The m.4435A>G mutation caused autophagic and mitophagic activity, promoting apoptosis
• Defective tRNA nucleotide modification impaired mitochondrial and cellular proteostasis

## Graphical abstract

**Figure:**
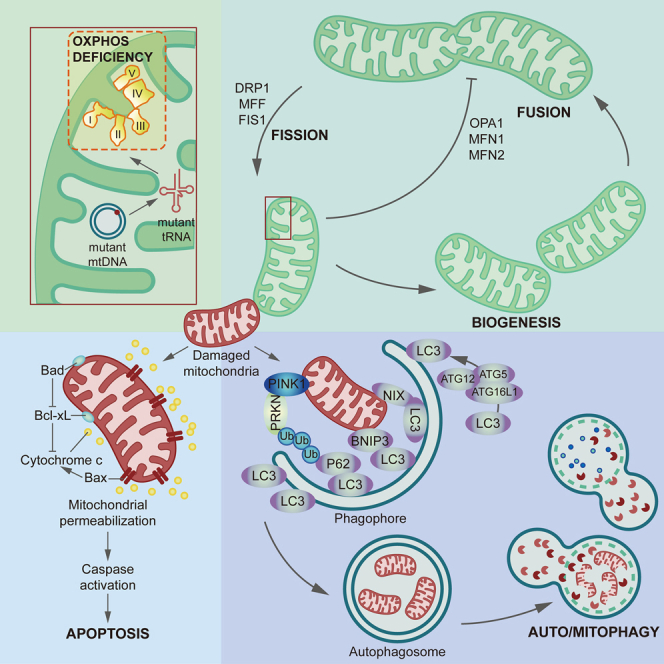

## Introduction

Mitochondria are the eukaryotic organelles not only for generating energy through the oxidative phosphorylation system (OXPHOS) but also acting as critical mediators of signals to propagate various cellular outcomes.^1^^,^^2^^,^^3^ Mitochondrial damages due to mitochondrial DNA (mtDNA) mutations are associated with a wide spectrum of clinical abnormalities.^1^ Health mitochondria in the cells are maintained by the processes of biogenesis, dynamics of fusion and fission as well as targeted degradation.^4^^,^^5^ Mitochondrial biogenesis requires the interplay between mitochondrial genes coding for 13 polypeptides for OXPHOS, 22 tRNAs and 2 rRNAs, and nuclear genes encoding approximately 1,500 mitochondrial proteins including 72 OXPHOS subunits, which are synthesized in cytosol and imported into mitochondria.^6^^,^^7^^,^^8^ In fact, the synthesis of 13 mtDNA encoding OXPHOS subunits is undertaken by own translation machinery composed of 2 rRNAs and 22 tRNAs and nucleus-encoding components such as ribosomal proteins and translation elongation factor TUFM.^9^^,^^10^ The fidelity and efficiency of translation were impacted by the nucleotide modifications at positions 34 and 37 at anticodon loop of mitochondrial tRNA (mt-tRNA).^11^^,^^12^^,^^13^^,^^14^^,^^15^ The deficient nucleotide modifications of mt-tRNAs impaired translation and mitochondrial functions.^15^^,^^16^^,^^17^^,^^18^^,^^19^ In fact, the organelles have their own defense pathways to respond to these genetic and environmental stressors and maintain their quality control via fusion and fission processes as well as mitophagy.^7^^,^^20^^,^^21^ In particular, these mitochondria dysfunctions due to mt-tRNA mutations may dysregulate the nuclear gene expression to cell function such as autophagy and apoptosis through mitochondrial retrograde signal pathways.^2^^,^^22^ However, the mechanism underlying deficient mt-tRNA posttranscription modification regulates the OXPHOS biogenesis, mitochondrial quality controls, and apoptosis is far less understood.

The modifications at position 37 (A or G) (adjacent to 3′ anticodon) of mt-tRNA play a vital role in the structure and function of mt-tRNA and mitochondrial functions.^11^^,^^12^^,^^13^^,^^17^^,^^18^ Our previous investigations showed that the A to G substitution at position 37 by the mt-tRNA^Ile^, mt-tRNA^Asp^, and mt-tRNA^Met^ affected the modification at this position and thereby perturbed the structure and function of tRNAs.^17^^,^^18^^,^^23^^,^^24^^,^^25^ In particular, the mt-tRNA^Met^ 4435A>G mutation-induced deficient nucleotide modification altered the mt-tRNA metabolism, codon-anticodon interactions, and led to faulty mitochondrial translation, especially in the fidelity and efficiency.^24^^,^^25^ The impairment of mitochondrial translation impeded the activities of OXPHOS complexes, diminished mitochondrial ATP levels, reduced membrane potential, and elevated the production of reactive oxygen species (ROS).^25^ The m.4435A>G mutation-induced deficiencies may dysregulate the expression of nucleus-encoding mitochondrial proteins involved in mitochondrial biogenesis, autophagy, and apoptosis. In this study, we demonstrated that the m.4435A>G mutation dysregulated the expression of nuclear genes encoding OXPHOS subunits and affected the assembly and biogenesis of OXPHOS complexes. We assessed if the instability and dysfunction of OXPHOS due to the m.4435A>G mutation then mediated the mitochondrial quality control processes via fission and fusion. We then examined if the dysregulation of mitochondrial quality control process regulated the signaling pathways involved in selective degradation of damaged mitochondria and cell death machinery intrinsic apoptosis.

## Result

### Dysregulation of nucleus-encoding subunits of OXPHOS

**Figure 1:**
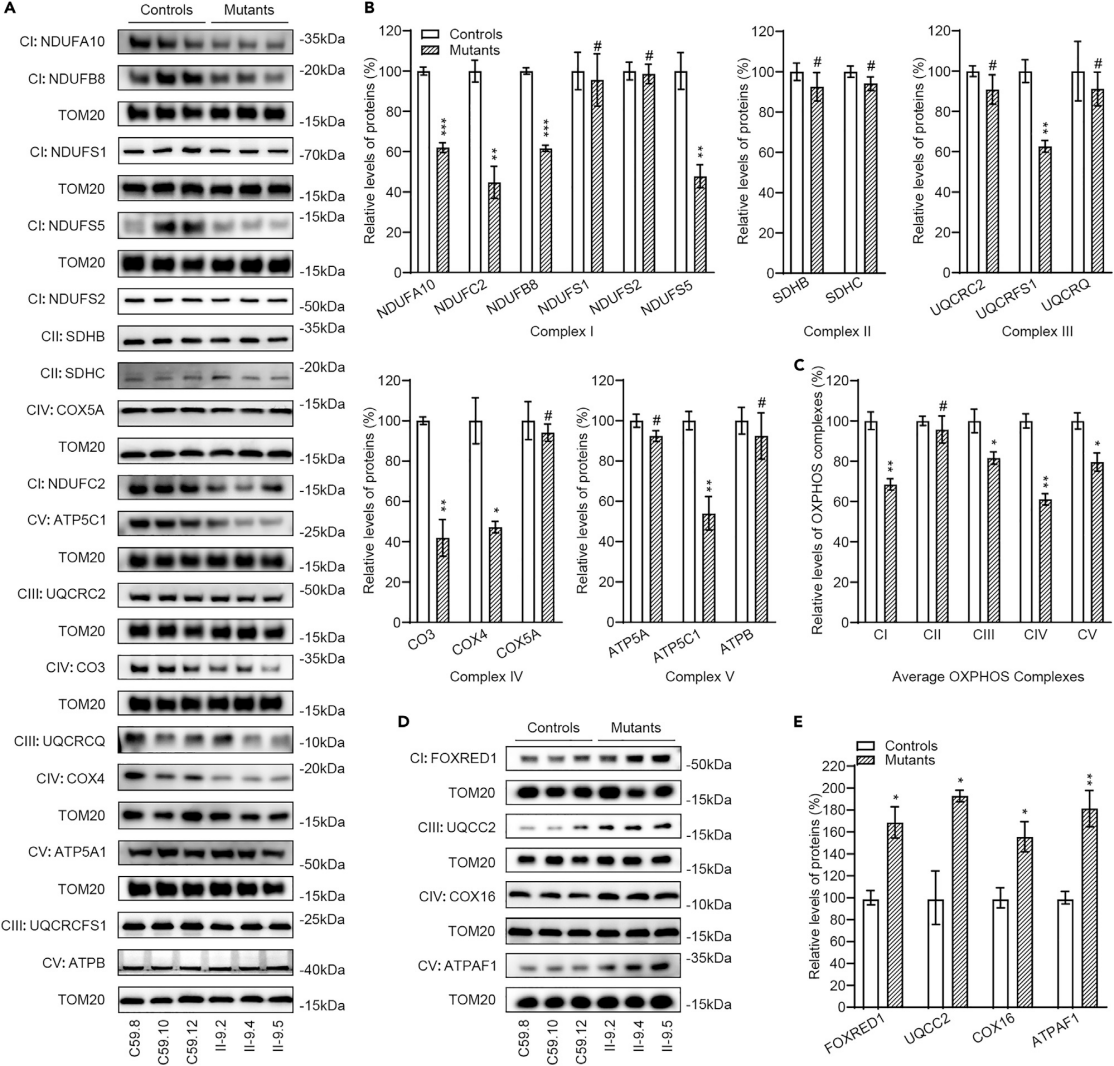
Western blot analysis of mitochondrial proteins (A and D) Twenty micrograms of total cellular proteins from various cell lines were electrophoresed through a denaturing polyacrylamide gel, electroblotted, and hybridized with antibodies for 17 subunits (CO3 encoded by mtDNA and 16 encoded by nuclear genes) (A) 4 assembly factors (D) of OXPHOS, and TOM20 as a loading control, respectively. (B and E) Quantification of 17 subunits (B) and 4 assembly factors (E) of OXPHOS. Average relative each polypeptide content per cell was normalized to the average content per cell of TOM20 in each cell line. The values for the latter are expressed as percentages of the average values for the control cell line. (C) Average levels of subunits from each complex of OXPHOS (6 of complexes I, 2 of II, 3 of III, 3 of IV, and 3 of V). The calculations were based on three independent determinations. The error bars indicate two standard error of the mean (SEM) of the means. p indicates the significance, according to the t test, of the differences between mutant and control cell lines. ∗p < 0.05; ∗∗p < 0.001; ∗∗∗p < 0.0001; #, not significant.

To assess the effect of m.4435A>G mutation on the OXPHOS biogenesis, we carried out the western blot analysis to examine the levels in 17 subunits of OXPHOS complexes in mutant and control cybrid cells using TOM20 as a loading control. These subunits included one mtDNA encoding polypeptide (CO3), 16 nucleus encoding proteins: NDUFC2, NDUFS1, NDUFS2, NDUFS5, NDUFA10, and NDUFB8 (subunits of complex I), SDHB and SDHC [subunits of succinate dehydrogenase (complex II)], UQCRFS1, UQCRQ, and UQCRC2 [subunits of ubiquinol-cytochrome c reductase (complex III)], COX5A and COX4 (subunits of complex IV), and ATP5C and ATP5A and ATPB [subunits of H^+^-ATPase (complex V)].^1^ As shown in Figures 1A and 1B, the significant decreases in the levels of 8 mitochondrial proteins (but not of NDUFS1, NDUFS2, SDHB, SDHC, UQCRQ, UQCRC2, COX5A, ATP5A, and ATPB) were observed in the mutant cell lines, as compared with the WT cells. As shown in Figure 1B, the levels of NDUFA10, NDUFC2, NDUFB8, NDUFS5, UQCRFS1, CO3, COX4, and ATP5C1 in mutant cell lines were 62%, 45%, 62%, 48%, 63%, 42%, 47%, and 54%, relative to the mean values measured in the control cell lines, respectively. Notably, the average levels in the subunits of complexes I, II, III, IV, and V in the mutant cell lines were 68%, 96%, 82%, 61%, and 80% of average values measured in the control cell lines, respectively (Figure 1C).

To test whether the m.4435A>G mutation affected the assembly of OXPHOS complexes, we measured the levels of assembly components (FOXRED1 for complex I, UQCC2 for complex III, COX16 for complex IV and ATPAF1 for complex V) in the mutant and control cell lines.^26^^,^^27^ As shown in Figures 1D and 1E, the levels of FOXRED1, UQCC2, COX16, and ATPAF1 in the mutant cell lines were 169%, 193%, 156, and 181% of those in the control cell lines, respectively. These indicated that m.4435A>G mutation dysregulated nucleus-encoding subunits of OXPHOS and impaired the assembly of OXPHOS complexes.

### Defective assembly and activity of OXPHOS complexes

**Figure 2:**
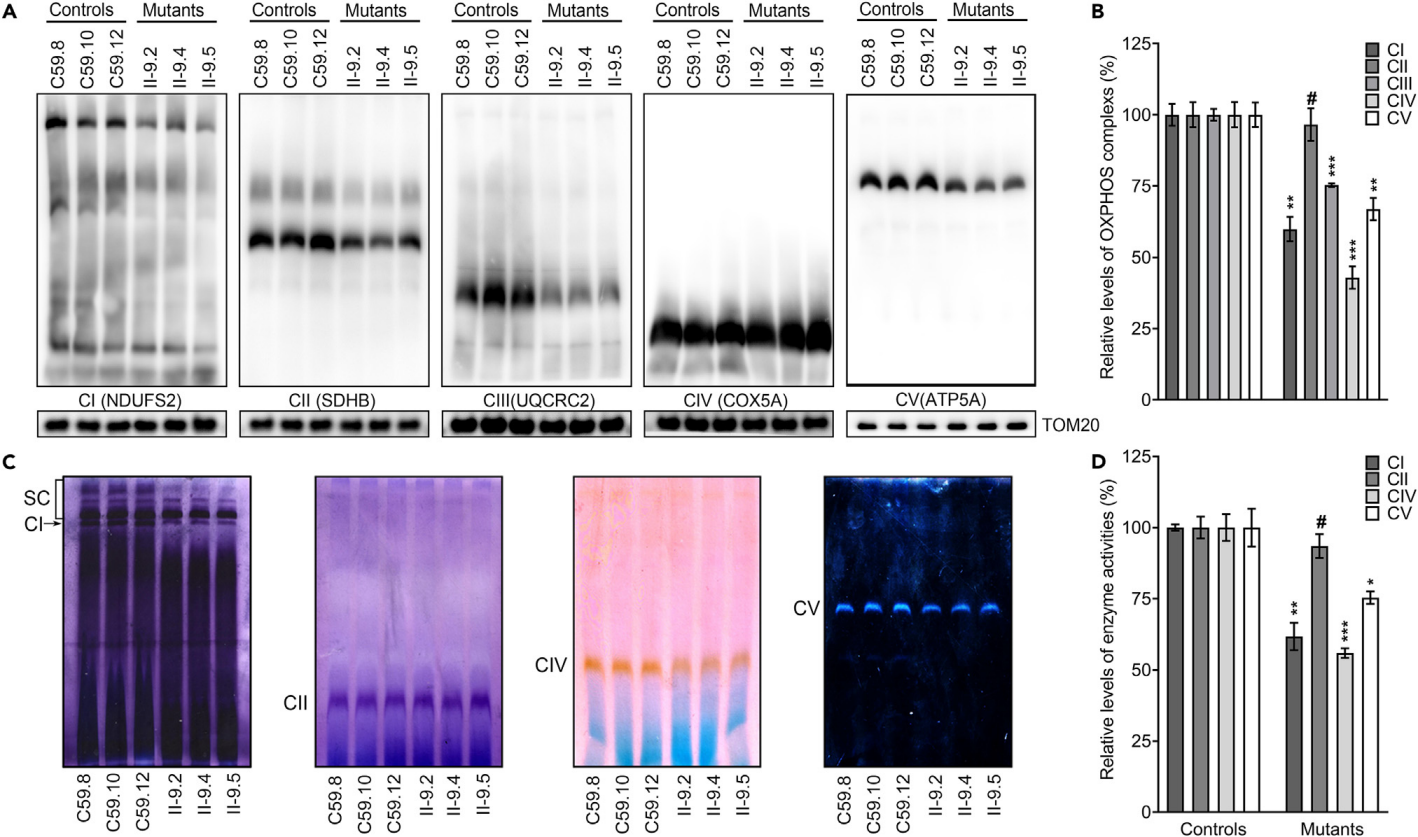
Defective assembly and activity of OXPHOS complexes (A) The steady-state levels of five OXPHOS complexes by blue native gel electrophoresis. Twenty micrograms of mitochondrial proteins from various cell lines were electrophoresed through a Blue Native gel, electroblotted, and hybridized with antibodies specific for subunits of five OXPHOS complexes (NDUFS2 for complex I, SDHB for complex II, UQCRC2 for complex III, COX5A for complex IV, and ATP5A a for complex V), and with TOM20 as a loading control. (B) Quantification of levels of complexes I, II, III, IV, and V in mutant and control cell lines. The calculations were based on three independent experiments. (C) In-gel activity of complexes I, II, IV, and V. The activities of OXPHOS complexes from various cell lines after BN-PAGE were measured in the presence of specific substrates [NADH and NTB for complex I, sodium succinate, phenazine methosulfate, and NTB for complex II, DAB and cytochrome *c* for complex IV, glycine, MgSO4, ATP, and Pb(NO3)2 for complex V].^16^ (D) Quantification of in-gel activities of complexes I, II, IV, and V. The calculations were based on three independent determinations in each cell line. Graph details and symbols are explained in the legend to Figure 1.

We examined the consequence of the m.4435A>G mutation-induced deficiency on the assembly and activities of OXPHOS complexes. Mitochondria isolated from various cell lines were analyzed by BN-PAGE and western blot analysis.^28^^,^^29^ As shown in Figure 2A, mutant cell lines bearing the m.4435A>G mutation displayed aberrant assembly of complexes I, III, IV, and V but not II. In particular, the average levels of complexes I, II, III, IV, and V in three mutant cell lines were 60%, 97%, 75%, 43%, and 67%, relative to the mean values measured in three control cell lines, respectively (Figure 2B).

We then assessed the stability and activities of complexes I, II, IV, and V using the in-gel activity assay. Mitochondrial membrane proteins isolated from various cell lines were separated by BN-PAGE and stained with specific substrates of complexes I, II, IV, and V.^29^^,^^30^ Defective assembly of complexes I, IV and V were further confirmed in the mutant cell lines, as compared with control cell lines (Figures 2C and 2D). In particular, the in-gel activities of complexes I, IV and V in mutant cell lines were 62%, 56%, and 75%, relative to the average values of control cell lines, respectively. In contrast, the in-gel activities of complexes II in the mutant cell lines were comparable with those of control cell lines.

### Imbalance of mitochondrial dynamics

**Figure 3:**
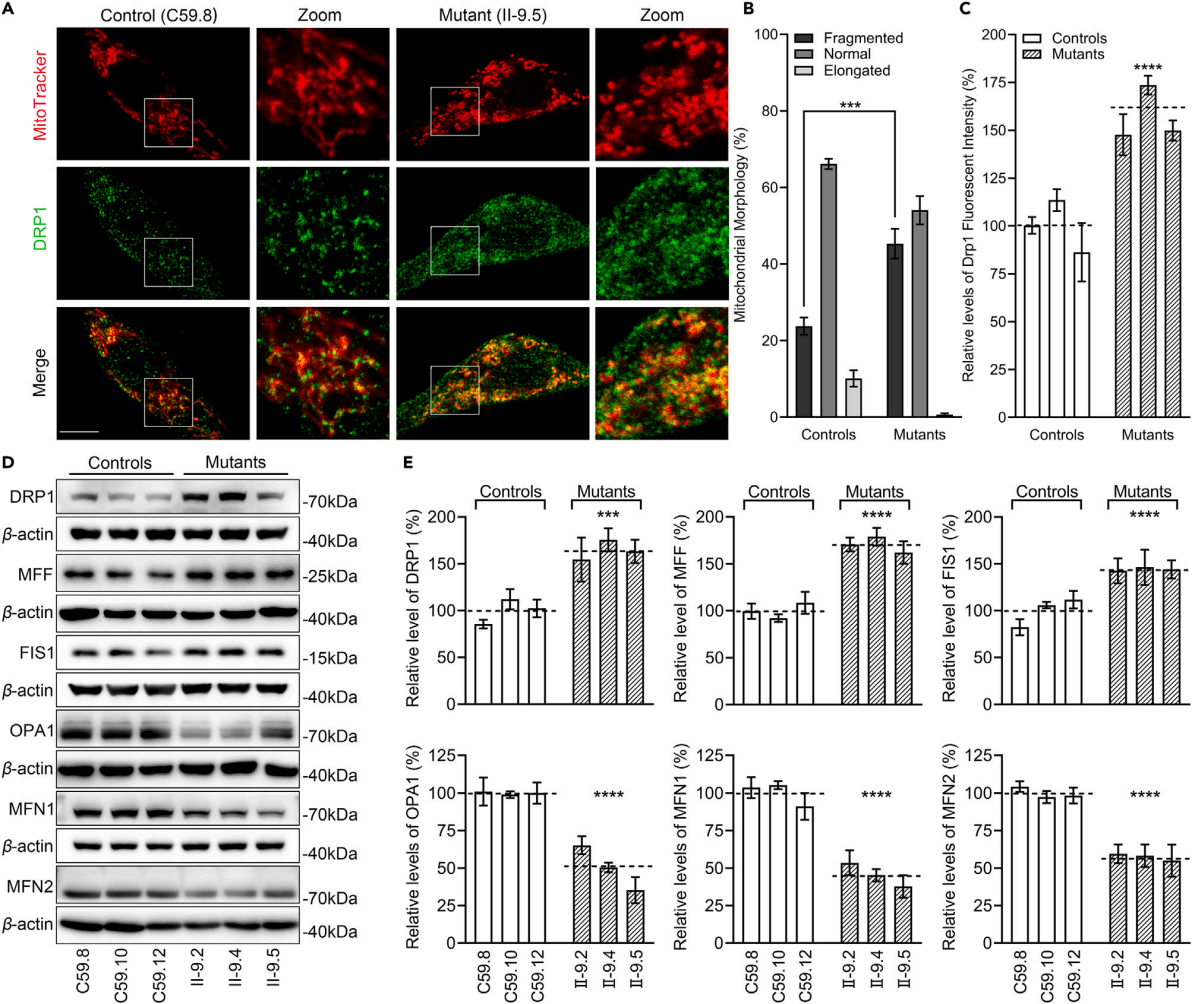
Assessment of mitochondrial dynamics (A) Immunofluorescence analysis. The distributions of cytochrome *c* from mutant II-9.5 and control C59.8 cybrids were visualized by immunofluorescent staining with mitochondrial dye MitoTracker and labeling with DRP1 antibody conjugated to Alex Fluor 488 (green) analyzed by confocal microscopy. Scale bars: 10 μm. (B) Quantification of mitochondrial morphology. Mitochondrial morphology was scored as follows: fragmented, mainly small and round; normal, mixture of round and shorter tubulated; and elongated, long and higher interconnectivity. The percentage of cells with indicated mitochondrial morphologies was determined as a percentage of the total number of cells counted (≥100 cells per experiment). n = 3 independent experiments. (C) Quantification of levels of DRP1 fluorescence intensity. Three independent determinations were done in each cell line. (D) Western blot analysis of mitochondrial fission-associated proteins (DRP1, MFF, and FIS1) and fusion-associated proteins (OPA1, MFN1, and MFN2) in six cell lines with *β*-actin as a loading control. (E) Quantification of mitochondrial fission-associated proteins (DRP1, MFF, and FIS1) and fusion-associated proteins (OPA1, MFN1, and MFN2). Three independent experiments were made for each cell line. Graph details and symbols are explained in the legend to Figure 1.

The m.4435A>G mutation-induced mitochondrial dysfunctions may affect the mitochondrial integrity and homeostasis, which is achieved through constant fusion and fission. We used immunofluorescence to assess the effect of m.4435A>G mutation on mitochondrial morphology and dynamics using cells staining with mitochondrial dye MitoTracker and labeling with antibody of fission-related protein Drp1. As shown in Figures 3A and 3B, mutant cells bearing the m.4435A>G mutation revealed abnormal mitochondrial morphologies, including markedly increased fragments and reduced elongated network of mitochondria, as compared with control cells. As shown in Figures 3A and 3C, the immunofluorescence patterns of double-labeled cells with rabbit monoclonal antibody specific for Drp1 and Mitotracker revealed 57% increases in the levels of Drp1 in the mutant cells, compared with control cells. This indicated that the m.4435A>G mutation promoted mitochondrial fission. The levels of Drp1 in mutant and control cell lines were further confirmed by western blot analysis (Figures 3D and 3F). Furthermore, we examined the levels of two fission-related proteins (MFF and FIS1) and three fusion-related proteins (OPA1, MFN1, and MFN2) in mutant and control cell lines by western blot analysis.^31^^,^^32^ As shown in Figures 3D–3F, mutant cell lines displayed elevating levels of fission-related proteins but reduced levels of fusion proteins. In particular, the average levels of MFF and FIS1 in three mutant cell lines were 171% and 144% of the average values measured in three control cell lines, respectively (Figure 3E). In contrast, the average levels of OPA1, MFN1, and MFN2 in three mutant cell lines were 50%, 46%, and 58% of the mean values measured in three control cell lines, respectively (Figure 3F). These data indicated that the m.4435A>G mutation led to the mitochondrial dynamic imbalance toward promoting fission.

### Promoted autophagy

**Figure 4:**
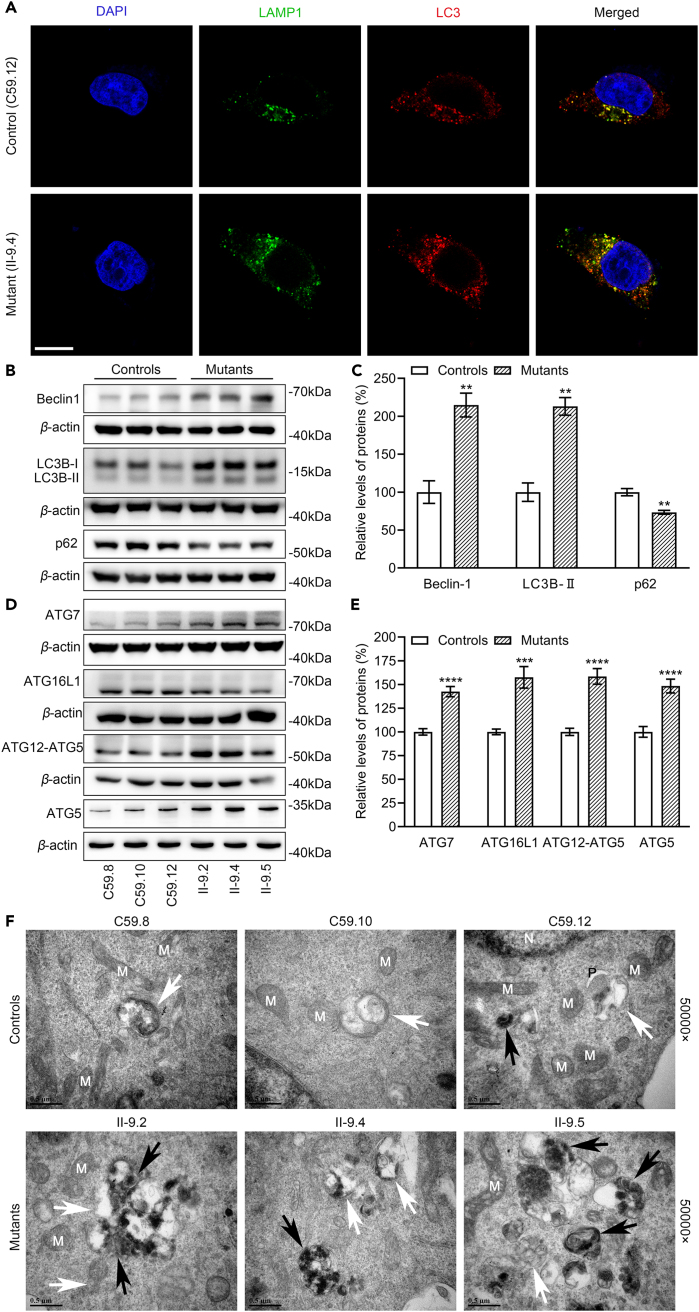
Analysis of autophagy (A) Immunofluorescence analysis. The distributions of LAMP1 from cybrids (C59.12 and II.9-4) were visualized by immunofluorescent labeling with LAMP1 antibody conjugated to Alex Fluor 488 (green) and LC3 antibody conjugated to Alex Fluor 594 (red) analyzed by confocal microscopy. DAPI-stained nuclei were shown by the blue fluorescence. Scale bars: 10 μm. (B and D) Western blot analysis of autophagy-associated proteins (B) and autophagosome formation and maturation associated proteins (D) in six cell lines with *β*-actin as a loading control. (C and E) Quantification of markers of autophagy (C) and autophagosome formation and maturation associated proteins (E). Three independent determinations were done in each cell line. (F) Cells from mutant and control cybrids were examined by transmission electron microscopy of initial autophagic vacuoles (white), degradative autophagic vacuole (black); M: mitochondria; N: nucleus; P: phagophore; scale bar: 500 nm. Ultrathin sections were stained with uranyl acetate and alkaline lead citrate. 50,000× magnifications were used. Graph details and symbols are explained in the legend to Figure 1.

**Figure 5:**
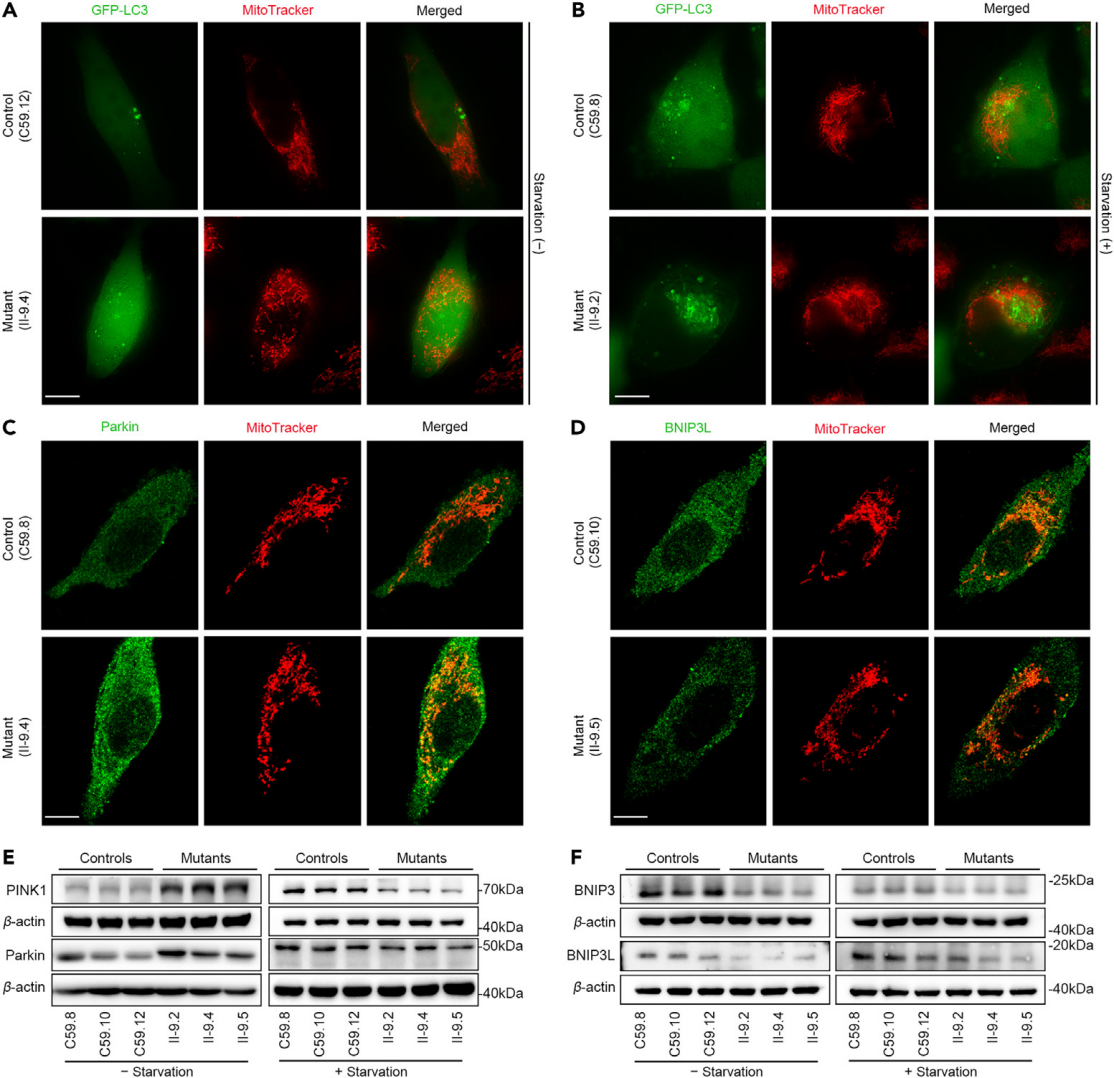
Mitophagy assays (A and B) Immunofluorescence assays using live cell imaging under normal or energy starved conditions in the absence (A) and presence (B) of HBSS. The distributions of LC3 from mutant and control cybrids were visualized by GFP-LC3 (green) and immunofluorescent staining with mitochondrial dye MitoTracker (red) analyzed by confocal microscopy. Scale bars: 10 μm. (C and D) Immunofluorescence analysis. The distributions of PARKIN and BNIP3L from mutant and control cybrids were visualized by immunofluorescent staining with mitochondrial dye MitoTracker (red) and labeling with PARKIN (C), and BNIP3L (D) antibody conjugated to Alex Fluor 488 (green) analyzed by confocal microscopy. (E and F) Western blot analysis of PARKINdependent mitophagy proteins (E) and ubiquitination-independent mitophagy proteins (F) in six cell lines with *β*-actin as a loading control.

Increased mitochondrial fission by the m.4435A>G mutation may facilitate autophagy. We assessed the autophagic states of mutant and control cell lines using immunoblotting and immunofluorescence assays. As shown in Figure 4A, mutant cell lines displayed markedly increases in the levels of lysosome-associated membrane glycoprotein 1 (LAMP1) and microtubule-associated protein 1A/1B light chain 3 (LC3), indicating that the m.4435A>G mutation significantly increased autophagy activity. The increasing levels of LC3 in the mutant cybirds were further confirmed by the autophagy flux using live cell imaging techniques (Figure S1). The effect of m.4435A>G mutation on autophagy was then examined by western blot analysis using three markers: LC3, P62, and Beclin-1, which involved in the initiation phase of autophagy, in mutant and control cell lines. During autophagy, the cytoplasmic form (LC3-I) is processed into a cleaved and lipidated membrane-bound form (LC3-II), which is recruited to autophagosomal membranes. The amount of LC3-II is clearly correlated with the number of autophagosomes.^33^ The sequestosome 1/P62 protein (SQSTM1, hereafter referred to P62), is an autophagy substrate that colocalizes with ubiquitinated protein aggregates.^34^ As shown in Figures 5B and 5C, increased levels of Beclin-1 and LC3, but decreased levels of P62 were observed in mutant cell lines carrying the m.4435A>G mutation, compared with control cell lines. In particular, the average levels of Beclin-1, LC3-II, and P62 in the three mutant cell lines carrying the m.4435A>G mutation were 215% (p = 0.0061), 213% (p = 0.0026), and 73% (p = 0.0079) of the mean values measured in three control cell lines, respectively.

To examine the effect of m.4435A>G mutation on the autophagosome formation and maturation, we measured the levels of ATG5, ATG7, ATG16L1, and ATG12 in the mutant and control cell lines using western blot analysis. In fact, the activity of the conserved Atg12–Atg5-Atg16 complex is essential for autophagosome formation and membrane elongation, while ATG7 plays a central role in autophagosome biogenesis by conjugating ATG5 to ATG12.^33^ As illustrated in Figures 4D and 4E, various increases in the levels of ATG5, ATG7, ATG16L1, and ATG12-ATG5 were observed in the mutant cybrids, as compared with those in control cybrids. In particular, the levels of ATG5, ATG7, ATG16L1, and ATG12-ATG5 in the mutant cybrids were 148%, 143%, 158%, and 158%, respectively. These results implicated that the m.4435A>G mutation promoted the formation and maturation of autophagosome.

To further investigate the process of autophagy, transmission electron microscopy was used to monitor the autophagy and quantification of autophagic accumulation in mutant and control cells. As shown in Figure 4F, the mutant cells exhibited predominant accumulations of matured late autophagic vacuoles than the formations of early autophagic vacuoles containing morphologically intact cytoplasm, as compared with those in the control cells. These data suggest that the m.4435A>G mutation impaired autophagy, especially the defects in the formation of autolysosome.

### Impaired mitophagy

Mitophagy is a specific form of autophagy that selectively removes damaged mitochondria by autophagosomes and their subsequent catabolism by lysosomes.^34^^,^^35^^,^^36^ Mitochondrial fragmentation resulting from decreased fusion and increased fission in mitochondria is necessary for mitophagy since smaller mitochondria are more easily engulfed by autophagosomes than larger ones and require less energy to be autophagocytosed. Based on the targeting signals on damaged or superfluous mitochondria that initiate mitophagy, this process can be grouped into ubiquitination-dependent mitophagy such as PARKIN dependent and ubiquitination-independent or receptor based mitophagy including apoptosis related proteins as mitophagy receptors or inhibitor.^34^ To investigate the effect of the m.4435A>G mutation on dynamics of mitophagy, we performed the immunofluorescence assays using live cell imaging under normal or energy starved (absence and presence of HBSS) conditions. As shown in Figures 5A and 5B, mutant cell lines displayed markedly increased or reduced levels of LC3 in mitochondria under normal and energy starved conditions, respectively, indicating that the m.4435A>G mutation impaired the mitophagy activity. To further investigate the impact of m.4435A>G mutation on mitophagy, we evaluated the mitophagic states of mutant and control cell lines using immunofluorescence and western blot assays with proteins involved in PARKIN dependent mitophagy and ubiquitination-independent mitophagy. Upon acute mitochondrial dysfunction, PARKIN dependent mitophagy (PINK1-PARKIN Pathway) is activated by PARKIN recruitment from the cytosol to the mitochondrial surface, ultimately leading to mitophagy.^36^^,^^37^ Furthermore, ubiquitination-independent mitophagy was regulated by pro-apoptotic proteins BNIP3 and BNIP3-like (BNIP3L) or Nip3-like protein X (NIX) belonging to the BCL2 family.^34^ Immunofluorescence assays revealed increasing levels of PARKIN but decreased levels of BNIP3L in mitochondria in the mutant cybrids, as compared with those in the control cybrids, respectively (Figures 5C and 5D). The levels of PARKIN, PINK1, BNIP3, and BNIP3L in mutant and control cell lines were further evaluated by western blot analysis under normal and energy starved conditions. As shown in Figures 5E and S2), the levels of PARKIN and PINK1 in mutant cybrids were markedly increased under normal condition but drastically decreased under energy starved condition, relative to the average values in three control cybrids, respectively. By contrast, the levels of BNIP3L in the mutant cybrids were markedly reduced in the mutant cybrids under normal and energy starved conditions (Figures 5F and S2). These data indicated that the m.4435A>G mutation upregulated the PARKIN dependent mitophagy but downregulated the ubiquitination-independent mitophagy.

### Upregulated intrinsic apoptosis

**Figure 6:**
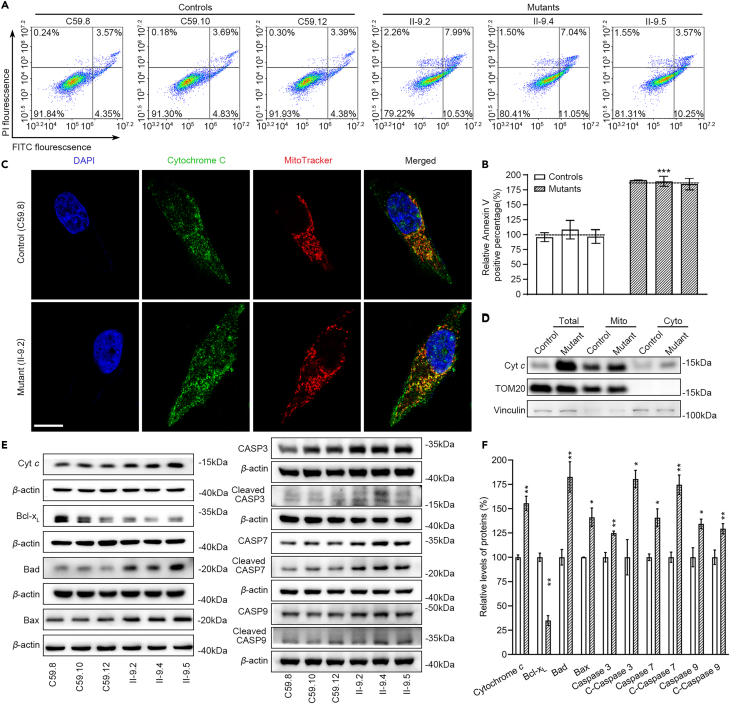
Upregulated intrinsic apoptosis (A) Annexin V/PI apoptosis assay by flow cytometry. Cells were harvested and stained with Annexin V and 1 μL of propidium iodide. The percentage of Annexin V-positive cells were assessed. (B) Relative Annexin V-positive cells from various cell lines. Three independent determinations were done in each cell line. (C) Immunofluorescence analysis. The distributions of cytochrome *c* from mutant II-9.2 and control C59.8 cybrids were visualized by immunofluorescent staining with mitochondrial dye MitoTracker and labeling with cytochrome *c* antibody conjugated to Alex Fluor 488 (green) analyzed by confocal microscopy. DAPI stained nuclei were identified by their blue fluorescence. Scale bars: 10 μm. (D) The levels of cytochrome *c* in cytosol in mutant and control cell lines were measured by fractioning the cells into mitochondrial and cytosolic fractions and western blot analysis using cytochrome *c*, Tom20 for mitochondrial protein and Vinculin for cytosolic protein. Total, total cell lysate; Cyto, cytosol; Mito, mitochondria. (E) Western blotting analysis of apoptosis-associated proteins. Total cellular proteins (20 μg) from various cell lines were electrophoresed, electroblotted and hybridized with several apoptosis-associated protein antibodies: cytochrome *c*, Bcl-xL, BAD, BAX, uncleaved caspases 9, caspases 3 and caspases 7, and cleaved caspases 3, 7, and 9, with *β*-actin as a loading control. (F) Quantification of apoptosis-associated proteins: cytochrome *c*, Bcl-xL, BAD, BAX, uncleaved caspases 3, 7, and 9, and cleaved caspases 3, 7, and 9. The levels of apoptosis-associated proteins in various cell lines were determined as described elsewhere.^41^ Three independent experiments were made for each cell line. Graph details and symbols are explained in the legend to Figure 1.

Mitochondrial fission machinery actively participates in the process of intrinsic apoptosis.^38^ The impact of m.4435A>G mutation-induced fission on apoptotic process was examined by Annexin V/PI-based flow cytometry for cellular apoptosis, immunofluorescence and western blot assays. As shown in Figures 6A and 6B, the average ratios of Annexin V-positive cells in the mutant cell lines carrying the m.4435A>G mutation were 188% of the mean values measured in the control cell lines. As shown in Figures 6C and 6D, the immunofluorescence patterns of double-labeled cells with rabbit monoclonal antibody specific for the cytochrome *c* and MitoTracker displayed markedly increased levels of cytochrome *c* in the mutant cells, compared with control cells. The levels of cytochrome *c* in cytosol in mutant and control cell lines were further evaluated by fractioning the cells into mitochondrial and cytosolic fractions and western blot analysis. As shown in Figures 6D–6F, the levels of cytochrome *c* in the mutant cell lines were markedly increased in the total cellular, mitochondrial and cytosolic fractions, as compared with those in control cell lines. Furthermore, we examined the levels of one apoptosis inhibited protein (Bcl-xL) and eight activated proteins (BAD, BAX, uncleaved/cleaved caspases 3, 7, and 9) in mutant and control cell lines by western blot analysis.^39^^,^^40^ As shown in Figures 6E and 6F, the average levels of Bcl-xL, BAD, BAX, uncleaved/cleaved-caspases 3, 7, and 9 in three mutant cell lines were 35%, 183%, 141%, 125%, 180%, 141%, 175%, 134%, and 129% of the mean values measured in three control cell lines, respectively. These results strongly indicated that the m.4435A>G mutation promoted apoptotic process.

## Discussion

The mechanism underlying mitochondrial tRNA mutations regulated the expression of nuclear genes to maintain healthy organelle populations for cellular function is far less understood. In this study, we investigated the effect of mt-tRNA^Met^ 4435A>G mutation on mitochondrial OXPHOS integrity and activity, and intracellular signaling related to mitochondrial morphology, autophagy and cell death. The m^1^G37 modification of mt-tRNA^Met^ created by m.4435A>G mutation caused the instability, deficient aminoacylation and faulty codon-anticodon interactions, and thereby impaired the synthesis of 13 mtDNA-encoding OXPHOS subunits, especially in the fidelity and efficiency.^24^^,^^25^ As a result, the mt-tRNA^Met^ 4435A>G mutation led to significant decline of mitochondrial functions associated with abnormalities in the respiratory chain and ATP synthesis, diminished membrane potentials and increased oxidative stress.^25^ In particular, the altered quality and quantity of these mtDNA encoding proteins may result in the imbalances between the increased levels of *de novo* protein synthesis and decreased folding capacity for the mtDNA- and nucleus-encoded OHPHOS subunits.^42^ In this study, mutant cells bearing the m.4435A>G mutation displayed various reductions in the nucleus encoding OXPHOS subunits (NDUFA10, NDUFC2, NDUFB8, NDUFS5, UQCRFS1, COX4, and ATP5C1 but not in other 8 subunits including 2 complex II subunits) and assemble factors of complexes I, III, IV, and V. These deficiencies gave rise to the abnormal assemble and instability of complexes I, III, IV, and V as well as intact supercomplexes observed in the mutant cell lines bearing the m.4435A>G mutation. Indeed, these mtDNA-encoded subunits appear to act as seeds for building new complexes, which requires nucleus-encoding subunit import and assembly with the assistance of assembly factors and assembly factors for complexes I, III, IV, and V.^26^^,^^43^ These defects yielded the reduced activities of complexes I, III, IV, and V but not complex II. Therefore, the m.4435A>G mutation dysregulated the expression of nucleus-encoding OXPHOS subunits and thereby impaired the assemble, stability, and activity of OXPHOS.

The m.4435A>G mutation-induced dysfunction of OXPHOS makes mitochondria undergo constant fission and fusion to repair damaged OXPHOS components, which allows segregation of damaged mitochondria via the fission process, exchange of materials between healthy mitochondria via the fusion process, and finally the elimination of damaged mitochondria via mitophagy.^37^ Electron microscopy studies showed the abnormalities of mitochondrial structures, including markedly increased fragments and reduced elongated network, and an imbalance between mitochondrial dynamics-related proteins in the mutant cells bearing the m.4435A>G mutation. The mutant cells carrying the m.4435A>G mutation revealed markedly increased expression of nucleus-encoding fission-related genes *DRP1*, *FIS1*, and *MFN* than these control cells lacking the mutation. By contrast, marked reductions in the levels of three fusion-related proteins (OPA1, MFN1, and MFN2) were observed in mutant cells harboring the m.4435A>G mutation. These strongly indicated that the m.4435A>G mutation dysregulated mitochondrial dynamics through promoting fission and reducing fusion.

Imbalance of mitochondrial dynamics and impaired membrane potentials by the m.4435A>G mutation regulated mitophagy which is a mitochondria-specific type of autophagy to dispose of damaged mitochondria. In this study, the cells bearing the m.4435A>G mutation exhibited predominant accumulations of matured late autophagic vacuoles and marked increases in the levels of LAMP1 and LC3,^44^^,^^45^ The m.4435A>G mutation impaired the process of autophagy, including the initiation phase, supported by increased levels of Beclin-1 but decreased levels of P62, formation and maturation of autophagosome, evidenced by raising levels of ATG5, ATG7, ATG16L1, and ATG12-ATG5 in the cells carrying the m.4435A>G mutation.^36^^,^^37^^,^^45^ The m.4435A>G mutation-induced autophagy dysregulation was consistent with increased autophagy in the cells carrying the mt-tRNA^Ile^ 4295A>G and mt-tRNA^Ala^ 5587T>C mutations,^18^^,^^46^ suggesting that altered quality and quantity of 13 mitochondrial proteins caused by mitochondrial tRNA mutations upregulated autophagy pathway. By contrast, reduced levels of only ND1 or ND6 protein due to m.3460G>A and m.14484T>C mutations downregulated autophagy process.^41^^,^^47^^,^^48^ In fact, mitophagy regulatory pathways are classified as ubiquitin-dependent or -independent pathways.^49^ Mitochondrial depolarization activates PINK1 to recruit ubiquitin ligase, PARKIN, to mitochondria, leading to clearance, while excessive mitochondrial fission leads to depolarization-independent mitophagy.^36^^,^^45^ Strikingly, the m.4435A>G mutation upregulated the PARKIN dependent mitophagy, evidenced by markedly increased levels in PARKIN and PINK1 in cells carrying the m.4435A>G mutation, in contrast with reduced levels in PARKIN and PINK1 in the cells m.3460G>A or m.14484T>C mutation.^41^^,^^47^ Furthermore, the m.4435A>G mutation downregulated the ubiquitination-independent mitophagy, suggested by the significant decreases in the BNIP3 and BNIP3L acting as mitophagy receptors and pro-apoptotic proteins in the mutant cell lines.^34^^,^^49^ These suggested that the m.4435A>G mutation upregulated mitophagy, especially ubiquitination-dependent pathway.

The m.4435A>G mutation-induced elevation of mitochondrial fusion and ROS production and diminished membrane potential promoted intrinsic apoptotic process for the removal of damaged cells. In this study, we showed that the m.4435A>G mutation conferred defects in the apoptosis, evidenced by 88% increased levels of Annexin V intensity. The elevated levels of pro-apoptotic proteins Bax and Bak, which translocate to mitochondria, raised the release of cytochrome *c* into the cytosol, evidenced by immunofluorescence and western blot assays in the cybrids. The release of cytochrome *c* promotes the activation of caspase-3, -7, and -9, which subsequently initiates cell death.^38^^,^^39^ The impact of m.4435A>G mutation on intrinsic apoptotic process was further supported by decreased levels of anti-apoptotic members including Bcl-xL and BNIP3L/NIX.^50^ These findings demonstrated that mitochondria dysfunctions due to the m.4435A>G mutation dysregulated the signaling pathways for mitochondrial dynamics, selective degradation of damaged mitochondria and intrinsic apoptosis.

In summary, we demonstrated that the mt-tRNA^Met^ 4435A>G mutation dysregulated the expression of nuclear genes encoding cytosolic proteins involved in OXPHOS and impaired the assembly and activities of OXPHOS complexes. These mitochondrial dysfunctions led to the mitochondrial dynamic imbalance toward fission, elevated mitophagy via ubiquitination-dependent pathway and finally promoted intrinsic apoptosis. The broad effect of the m.44534A>G mutation on cytosolic signaling related to the mitochondrial and cellular integrity may regulate various aspects of vascular function, thereby being critical for the pathogenesis of hypertension. Thus, our findings may provide new insights into the understanding of the pathophysiology of maternally inherited hypertension.

### Limitations of the study

In this study, we highlight that deficient tRNA^Met^ modification dysregulated the expression of nuclear genes involved in oxidative phosphorylation system (OXPHOS) and impaired the assemble and integrity of OXPHOS complexes. These mitochondrial dysfunctions caused mitochondrial dynamic imbalance, impaired the process of autophagy including initiation phase, formation, and maturation of autophagosome. Strikingly, the m.4435A>G mutation upregulated the PARKIN dependent mitophagy pathways but downregulated the ubiquitination-independent mitophagy. However, the relationship and molecular mechanism between mitochondrial and cytosolic dysfunction remain unclear. These alterations promoted intrinsic apoptotic process for the removal of damaged cells, but the underlying mechanisms remain unclear. The potential caveats of the experimental approach to analyze mitochondrial dynamics were not high-resolution imaging of mitochondria. The mitochondrial retrograde signal pathways for mt-tRNA mutation regulating cytosolic signaling related to the mitochondrial and cellular integrity need to be further investigated.

## STAR★Methods

### Key resources table

**Table:** 

REAGENT or RESOURCE	SOURCE	IDENTIFIER
**Antibodies**		

Mouse monoclonal anti-LAMP1	Abcam	Cat#ab25630; RRID: AB_470708
Mouse monoclonal anti-SQSTM1/P62	Abcam	Cat#ab56416; RRID: AB_945626
Rabbit monoclonal anti-SDHC	Abcam	Cat#ab155999; RRID: AB_2810989
Mouse monoclonal anti-ATG16L1	Abcepta	Cat#AM1817b; RRID: AB_2227476
Mouse monoclonal anti-PINK1	Abcepta	Cat#AW5456; RRID: AB_2924295
Rabbit polyclonal anti-ATG12	Abcepta	Cat#AP1816a; RRID: AB_2059363
Rabbit polyclonal anti-ATG7	Abcepta	Cat#AP1813c; RRID: AB_2062350
Rabbit polyclonal anti-BECLIN1	Abcepta	Cat#AP1818b; RRID: AB_2064335
Rabbit monoclonal anti-*β*-Actin	Abclonal	Cat#AC038; RRID: AB_2863784
Rabbit polyclonal anti-ATG5	Abclonal	Cat#A0203; RRID: AB_2757017
Rabbit polyclonal anti-NDUFA10	Abclonal	Cat#A10123; RRID: AB_2757648
Rabbit polyclonal anti-NDUFC2	Abclonal	Cat#A15073; RRID: AB_2861711
Rabbit polyclonal anti-NDUFS2	Abclonal	Cat#A12858; RRID: AB_2861678
Rabbit polyclonal anti-NDUFS5	Abclonal	Cat#A1265; RRID: AB_2759495
Rabbit polyclonal anti-OPA1	Abclonal	Cat#A9833; RRID: AB_2770723
Rabbit polyclonal anti-PRKN/Parkin	Abclonal	Cat#A0968; RRID: AB_2757487
HRP-labeled goat anti-rabbit IgG	Beyotime	Cat#A0208; RRID: AB_2892644
HRP-labeled goat anti-mouse IgG	Beyotime	Cat#A0216; RRID: AB_2860575
Rabbit monoclonal anti-CASP7	Cell Signaling	Cat#12827; RRID: AB_2687912
Rabbit polyclonal anti-BCL-XL	Cell Signaling	Cat#2762; RRID: AB_10694844
Mouse monoclonal anti-CYTC	Proteintech	Cat#66264-1-Ig; RRID: AB_2716798
Mouse monoclonal anti-MFF	Proteintech	Cat#66527-1-Ig; RRID: AB_2881890
Mouse monoclonal anti-Vinculin	Proteintech	Cat#66305-1-Ig; RRID: AB_2810300
Rabbit polyclonal anti-ATP5A1	Proteintech	Cat#14676-1-AP; RRID: AB_2061761
Rabbit polyclonal anti-ATP5C1	Proteintech	Cat#10910-1-AP; RRID: AB_2877740
Rabbit polyclonal anti-ATPAF1	Proteintech	Cat#18016-1-AP; RRID: AB_2243352
Rabbit polyclonal anti-ATPB	Proteintech	Cat#17247-1-AP; RRID: AB_2061878
Rabbit polyclonal anti-BAD	Proteintech	Cat#10435-1-AP; RRID: AB_2061994
Rabbit polyclonal anti-BAX	Proteintech	Cat#50599-2-Ig; RRID: AB_2061561
Rabbit polyclonal anti-CASP3/P17/P19	Proteintech	Cat#19677-1-AP; RRID: AB_10733244
Rabbit polyclonal anti-CASP9/P35/P10	Proteintech	Cat#10380-1-AP; RRID: AB_2068632
Rabbit polyclonal anti-COX16	Proteintech	Cat#19425-1-AP; RRID: AB_10666854
Rabbit polyclonal anti-COX5A	Proteintech	Cat#11448-1-AP; RRID: AB_2085429
Rabbit polyclonal anti-DRP1	Proteintech	Cat#12957-1-AP; RRID: AB_2093525
Rabbit polyclonal anti-FIS1	Proteintech	Cat#10956-1-AP; RRID: AB_2102532
Rabbit polyclonal anti-FOXRED1	Proteintech	Cat#24595-1-AP; RRID: AB_2879629
Rabbit polyclonal anti-LC3B	Proteintech	Cat#18725-1-AP; RRID: AB_2137745
Rabbit polyclonal anti-MFN1	Proteintech	Cat#13798-1-AP; RRID: AB_2266318
Rabbit polyclonal anti-MFN2	Proteintech	Cat#12186-1-AP; RRID: AB_2266320
Rabbit polyclonal anti-NDUFB8	Proteintech	Cat#14794-1-AP; RRID: AB_2150970
Rabbit polyclonal anti-NDUFS1	Proteintech	Cat#18443-1-AP; RRID: AB_10699875
Rabbit polyclonal anti-SDHB	Proteintech	Cat#10620-1-AP; RRID: AB_2285522
Rabbit polyclonal anti-TOM20	Proteintech	Cat#11802-1-AP; RRID: AB_2207530
Rabbit polyclonal anti-UQCC2	Proteintech	Cat#25781-1-AP; RRID: AB_2880237
Rabbit polyclonal anti-UQCRC2	Proteintech	Cat#14742-1-AP; RRID: AB_2241442
Rabbit polyclonal anti-UQCRQ	Proteintech	Cat#14975-1-AP; RRID: AB_2288356
Rabbit polyclonal anti-UQCRFS1	Proteintech	Cat#18443-1-AP; RRID: AB_10699875
Rabbit polyclonal anti-CO3	Proteintech	Cat#55082-1-AP; RRID: AB_2881265
Rabbit polyclonal anti-BNIP3	Sangon Biotech	Cat#D121876
Mouse monoclonal anti-NIX	Santa Cruz	Cat#sc-166332; RRID: AB_2066782
Alexa Fluor 488 goat anti-mouse IgG	Thermofisher	Cat#A21202; RRID: AB_141607
Alexa Fluor 488 goat anti-rabbit IgG	Thermofisher	Cat#A11008; RRID: AB_143165
Alexa Fluor 594 goat anti-rabbit IgG	Thermofisher	Cat#A11012; RRID: AB_2534079

**Chemicals, peptides, and recombinant proteins**		

Nitrotetrazolium blue chloride	Sangon Biotech	A610379; CAS: 298-83-9
Sodium succinate	Sangon Biotech	A610889; CAS: 150-90-3
Phenazine methosulfate	Sangon Biotech	A610361; CAS: 299-11-6
DAB	Sangon Biotech	A600140; CAS: 868272-85-9
ATP·Na_2_	Sangon Biotech	A600020; CAS: 34369-07-8
Fluoromount	Sigma-Aldich	Cat#F4680
Phenylmethanesulfonyl fluoride	Sigma-Aldich	P7626; CAS: 329-98-6
Digitonin	Sigma-Aldich	D141; CAS: 11024-24-1
Chloroquine	Sigma-Aldich	C6628; CAS: 50-63-5
NADH	Roche	10107735001; CAS: 606-68-8
Cytochrome c	Sigma-Aldich	C2506; CAS: 9007-43-6
Triton X-100	Sigma-Aldich	T9284; CAS: 9036-19-5
Tween 20	Sigma-Aldich	P7949; CAS: 9005-64-5
MitoTracker Red CMXRos	Thermofisher	Cat#M7512
TEMED	Thermofisher	17919; CAS: 110-18-9
DAPI	Thermofisher	62248; CAS: 28718-90-3
Hoechst 33342	Thermofisher	Cat#H3570
HBSS	Thermofisher	Cat#14175095
Hieff Trans® Liposomal Transfection Reagent	Yeason	Cat#40802ES

**Critical commercial assays**		

BCA assay	Beyotime	Cat#P0009
Annexin V-FITC Apoptosis Detection Kit	Beyotime	Cat#C1062

**Deposited data**		

Raw and analyzed data	This paper	Mendeley Data: https://doi.org/10.17632/gbrw4vc99r.1

**Experimental models: Cell lines**		

	Zhou et al.^25^	N/A

**Recombinant DNA**		

pCMV_GFP-LC3	This paper	N/A

**Software and algorithms**		

Prism 9	GraphPad	https://www.graphpad.com/
ImageJ	NIH	https://imagej.nih.gov/ij/

### Resource availability

#### Lead contact

Further information and requests for resources and reagents should be directed to and will be fulfilled by the lead contact, Dr. Min-Xin Guan (gminxin88@zju.edu.cn).

#### Materials availability

This study did not generate new unique reagents.

#### Data and code availability

• Original Western blot images have been deposited at Mendeley and are publicly available as of the date of publication. The DOI is listed in the key resources table. Microscopy data reported in this paper will be shared by the lead contact upon request.
• This study did not generate any unique code.
• Any additional information required to reanalyze the data reported in this work paper is available from the lead contact upon request.

### Experimental model and study participant details

#### Cell lines and culture conditions

Three mutant cybrids (II-9.2, II-9.4, and II-9.5) bearing the m.4435A>G mutation and three control cybrids (C59.8, C59.10, and C59.12) belonging to the same mtDNA haplogroup but lacking the mutation were generated as described previously.^25^ All cell lines were grown in Dulbecco’s Modified Eagle Medium (DMEM) (Gibco) (containing 4.5 mg of glucose, 0.11 mg pyruvate/ml and 50 μg of uridine/ml), supplemented with 10% fetal bovine serum (FBS)(Gibco).

### Method details

#### Western blot analysis

Western blot analysis was performed as described elsewhere.^51^ Briefly, 20 μg of total cellular proteins obtained from various cell lines were denatured and electrophoresed through 10% bis-Tris SDS-polyacrylamide gels. Afterward, the gels were electroblotted onto polyvinylidene difluoride (PVDF) membrane for hybridization. Membranes were blocked in Tris-Buffered Saline and Tween20 (TBST) (150 mM NaCl, 10 mM Tris–HCl, pH 7.5 and 0.1% Tween 20) containing 5% (w/v) milk, then incubated with the corresponding primary and secondary antibodies. The primary antibodies used for this investigation were as described in the key resources table. Peroxidase Affinipure goat anti-mouse IgG and goat anti-rabbit IgG (Beyotime) were used as secondary antibodies, and protein signals were detected using Enhanced chemiluminescence system (CWBIO).

#### Blue native polyacrylamide gel electrophoresis and in-gel activity assays

BN-PAGE was performed on mitochondrial protein extracted from various cell lines as detailed elsewhere.^28^^,^^29^ For in-gel activity assays, samples containing 30 μg of total mitochondrial proteins were separated on 3 to 12% Bis-Tris Native PAGE gel. The native gels were prewashed in cold water and then incubated with the specific substrates of OXPHOS [NADH and NTB for complex I, sodium succinate, phenazine methosulfate, and NTB for complex II, DAB and cytochrome c for complex IV, glycine, MgSO_4_, ATP and Pb(NO_3_)_2_ for complex V] at room temperature.^16^^,^^29^ After stopping reaction with 10% acetic acid, gels were washed with water and scanned to visualize the activities of respiratory chain complexes.

#### Immunofluorescence analysis

Immunofluorescence experiments were undertaken as described elsewhere.^40^ Cells were cultured on cover glass slips (Thermofisher), fixed in 4% formaldehyde for 15 min, permeabilized with 0.2% Triton X-100, blocked with 5% FBS for 1 h, and immune-stained with DRP1, Cytochrome *c*, LC3B, LAMP1, Parkin, and BNIP3L antibodies overnight at 4°C, respectively. The cells were then incubated with Alex Fluor 594 goat anti-rabbit IgG, Alex Fluor 488 goat anti-rabbit IgG and Alex Fluor 488 goat anti-mouse IgG (Thermofisher), stained with 4′, 6-diamidino-2-phenylindole (DAPI; Thermofisher) for 15 min and mounted with Fluoromount (Sigma-Aldrich). Cells were examined using a confocal fluorescence microscope (Olympus Fluoview FV1000) with three lasers (Ex/Em = 550/570, 492/520, and 358/461 nm).

#### Transmission electron microscopy (TEM)

The cell lines were washed with PBS and fixed in 2.5% glutaraldehyde in phosphate buffer (0.1 M, pH 7.0) for 4 h and post-fixed with 1% OsO_4_ in phosphate buffer (0.1 M, pH 7.0) for 2 h. The samples were dehydrated with increasing concentrations of ethanol (30, 50, 70, 80, 90, 95 and 100) and transferred to absolute acetone for 20 min. After placing in 1:1 mixture of absolute acetone and the final Spurr resin (SPI-CHEM, 02690-AB) mixture for 1 h at room temperature, the samples were transferred to 1:3 mixture of absolute acetone and the final resin mixture for 3 h and to final Spurr resin mixture for overnight. Electron photomicrographs were taken from ultrastructures of cells under a transmission electron microscopy (Hitachi, H-7650).

#### Annexin V/PI apoptosis assay by flow cytometry

For discrimination of apoptotic and non-apoptotic cells by Annexin V/PI staining, cells were harvested and stained with Annexin V and 1 μL of propidium iodide (PI) (Beyotime) according to the manufacturer’s instruction. Each sample was detected by NovoCyte (ACEA Biosciences) and analyzed using NovoExpress software.^46^^,^^52^

### Quantification and statistical analysis

All statistical analyses were performed using GraphPad Prism (version 9.00) for statistical analysis to compare outcomes using a two-tailed unpaired Student’s *t* test. p values of less than 0.05 were considered to be statistically significant. ∗p < 0.05; ∗∗p < 0.001; ∗∗∗p < 0.0001; #, not significant.
