# Supplementary material for: Deficient tRNA posttranscription modification dysregulated the mitochondrial quality controls and apoptosis
Source: iScience. 2024 Jan 12;27(2):108883. doi: 10.1016/j.isci.2024.108883 (PMC10838789; doi:10.1016/j.isci.2024.108883)
Supplement: Document S1. Figures S1 and S2 [file mmc1.pdf]

iScience, Volume *27*

Supplemental information

Deficient tRNA posttranscription modification dysregulated the mitochondrial quality controls and apoptosis

Yunfan He, Gao Zhu, Xincheng Li, Mi Zhou, and Min-Xin Guan

SUPPLEMENTARY INFORMATION

Supplemental Figures S1 and S2

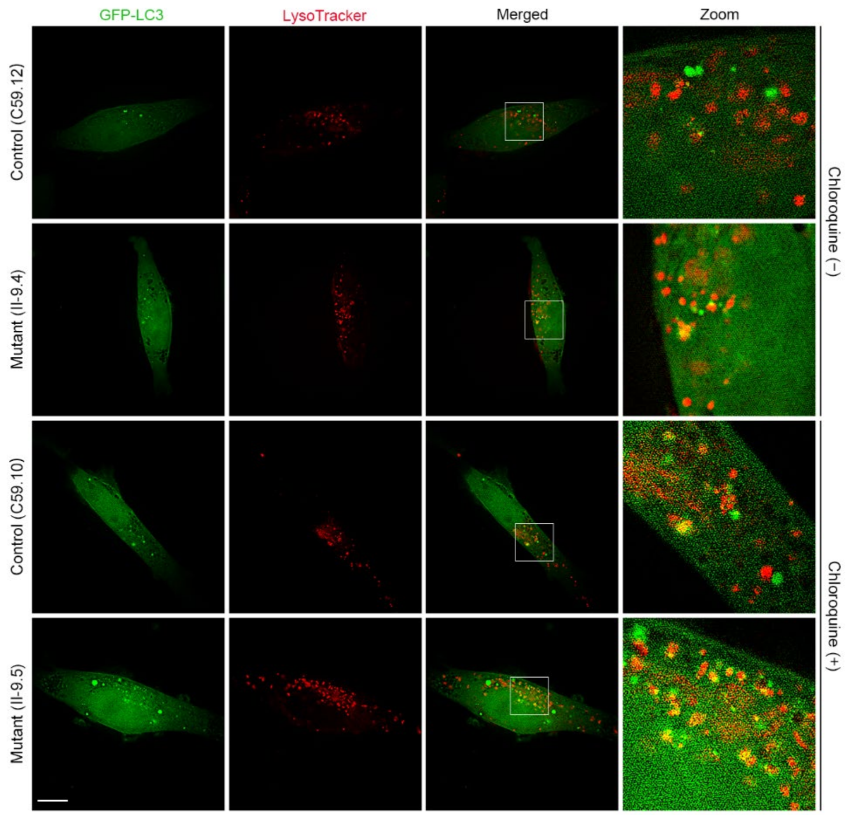

Supplemental Figure S1. Assay for the autophagy flux, related to Figure 4

Assay for the autophagy flux using live cell imaging in the absence (A) and presence (B) of Chloroquine 50µM. The distributions of LC3 from mutant and control cybrids were visualized by GFP-LC3 (green) and immunofluorescent staining with lysosome dye LysoTracker (red) analyzed by confocal microscopy. Scale bars: 10 µm.

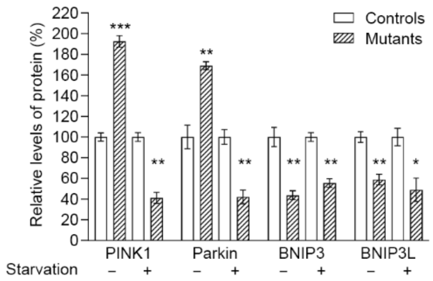

Supplemental Figure S2. Quantification of Parkin-dependent and ubiquitination-independent mitophagy proteins, related to Figure 5. The calculations were based on three independent determinations. The error bars indicate two standard error of the mean (SEM) of the means. *p* indicates the significance, according to the t-test, of the differences between mutant and control cell lines. *$P < 0.05$; **$P < 0.001$; ***$P < 0.0001$; #, not significant.
